# Impact of mouse strain and sex when modeling radiation necrosis

**DOI:** 10.1186/s13014-020-01585-5

**Published:** 2020-06-03

**Authors:** Andrew J. Boria, Carlos J. Perez-Torres

**Affiliations:** 1 School of Health Sciences, Purdue University, 550 Stadium Mall Drive, Hampton Hall 1263A, West Lafayette, IN USA; 2 Purdue University Center for Cancer Research, Purdue University, West Lafayette, IN USA

**Keywords:** Radiation necrosis, Sex as a biological variable, Mouse strain, Animal models

## Abstract

**Background:**

Murine models are among the most common type of preclinical animal models used to study the human condition, but a wide selection of different mice is currently in use with these differences potentially compromising study results and impairing the ability to reconcile interstudy results. Our goal was to determine how the strain and sex of the mice selection would affect the development of radiation necrosis in our murine model of radiation-induced cerebral necrosis.

**Methods:**

We generated this model by using a preclinical irradiator to irradiate a sub-hemispheric portion of the brain of mice with single-fraction doses of 80 Gy. Eight possible combinations of mice made up of two different with two substrains each (BALB/cN, BALB/cJ, C57BL/6 N, and C57BL/6 J) and both sexes were irradiated in this study. Radiation necrosis development was tracked up to 8 weeks with a 7 T Bruker MRI utilizing T2-weighted and post-contrast T1-weighted imaging. MRI results were compared to and validated with the use of histology which utilized a scale from 0 to 3 in ascending order of damage.

**Results:**

Both time post-irradiation and strain (BALB/c vs C57BL/6) were significant factors affecting radiation necrosis development. Sex was in general not a statistically significant parameter in terms of radiation necrosis development.

**Conclusion:**

Mouse strain thus needs to be considered when evaluating the results of necrosis models. However, sex does not appear to be a variable needing major consideration.

## Background

Mouse models of disease remain a powerful tool in biomedical research, with similar models being used across multiple labs spanning multiple countries. Ideally, these mouse models should produce results that are as close to identical as possible across all locations. However, for this to happen, the parameters used in each of the models should be as consistent as possible. That being said, some parameters will affect each model more than others. We are interested in modeling cerebral radiation necrosis, a consequence of brain irradiation for cancer that affects 3 to 23% of patients [1, 2]. Previously, we have shown how changes in the radiation delivery parameters, including a comparison of two radiation delivery devices, can affect the modeling of radiation necrosis (RN) in the mouse [3, 4].

Given that this is a model of a radiation-induced pathology, the natural inclination would be to focus primarily on replication of the radiation delivery parameters to ensure the model is reproducible. However, biological parameters can also influence the ability to properly reproduce the model. There are examples in the literature of brain irradiations being modeled in BALB/c [3, 4] and C57BL/6 [5, 6] mice, which is not surprising as they are the two most common “normal” mouse strains. Ironically, for the cited reports above, the BALB/c studies were done in females while the C57BL/6 studies were done in males which is another biological factor that could influence the response of the model. We know from studies on radiation lethality that BALB/c mice should be more sensitive to radiation that C57BL/6 [7, 8] with studies such as Okayasu et al. [9] explaining in depth the deficiencies in DNA repair of the former, however little to no research has been performed to validate this in a model of radiation necrosis.

The goal of this study was to determine the effect mouse strain and sex have in reference to our previously established model of RN [3, 4]. As both the BALB/c and C57BL/6 strains have existed for over 100 years, and genetic drift is known to have occurred [10, 11], we further decided to include two different vendors for each strain encompassing the N and J substrains for each strain. Radiation parameters were kept constant across all mice and consistent with our prior work. Consistent with the prior reports on radiosensitivity based on lethality, we found that BALB/c mice are more sensitive than C57BL/6 mice leading to accelerated onset of the model. However, sex was not found to affect the results of the model.

## Methods

All animal experiments were approved by the Purdue Animal Care and Use Committee which stipulated that any mouse that lost ≥20% of its starting weight at the time of irradiation had to be sacrificed for humane reasons. However, it must be noted that none of the mice had significant weight loss throughout the experiment. Irradiation of mice was followed by MRI at multiple timepoints to track radiation necrosis lesion progression followed by post-mortem validation with histology after the final timepoint.

### Setup and treatment

In depth details of our irradiation setup can be found in our prior publication [3]. Briefly, an X-Rad 320 (Precision X Ray, North Branford, CT) pre-clinical cabinet irradiator was used to deliver partial cerebrum doses to mice to a 0.5 cm by 0.5 cm field at a dose rate of about 2 Gy per minute. All mice received 80 Gy in a single treatment under isoflurane anesthesia, which was found to be an ideal dose for generating radiation necrosis in mice without having significant lethality concerns based on our previous work, which also found that single fraction doses of 50 Gy were insufficient to cause radiation necrosis within a time span of 26 weeks [3].

### Mouse strain and numbers

8–9-week-old mice were sourced as follows: BALB/cN (Harlan Laboratories Inc., Indianapolis, IN), BALB/cJ (Jackson Laboratory, Bar Harbor, ME), C57BL/6 N (Charles River Laboratories Inc., Wilmington, MA), and C57BL/6 J (Jackson Laboratory, Bar Harbor, ME). 95 total mice were used in this study: 15 female BALB/cN mice; 10 male BALB/cN mice; 15 female BALB/cJ mice; 10 male BALB/cJ mice; 15 female C57BL/6 N mice; 10 male C57BL/6 N mice; 10 female C57BL/6 J mice; and 10 male C57BL/6 J mice. Of these, 40 mice (5 of each of the 8 possible combinations of sex and substrain) were followed for 4-weeks, and 55 mice were followed for 8-weeks (2 died early).

### Magnetic resonance imaging (MRI)

Prior to imaging, inhaled isoflurane was used to anaesthetize mice, and mice were given an intraperitoneal injection of 0.2 mL of Multihance (gadobenate dimeglumine; Bracco Diagnostics Inc., Princeton, NJ) diluted to a 1:10 ratio in saline. Imaging was carried out using a Bruker BioSpec 70/30USR 7 T MRI (Billerica, MA) which was used to image mice at timepoints of 4, 6, and 8 weeks. Both RARE T2-weighted images (Effective TE = 40 ms, TR = 4000 ms, Averages = 4) and MSME T1-weighted images (TE = 8 ms, TR = 500 ms, Averages = 4) were acquired. Twenty-one slices with a 0.5 mm slice thickness were obtained for each scan type with the 3rd slice of both set of scans centered on where the olfactory bulbs and the rest of the cerebrum were separated. The matrix size of the scans was 128 pixels by 128 pixels with a field size of 15 by 15 mm^2^, with a corresponding resolution of ~ 0.117 mm.

### MRI data analysis

Quantification of the radiation necrosis lesion was carried out using a semi-automatic threshold segmentation algorithm as we have previously described [3]. The lesion was defined as the areas of both hyperintensity and hypointensity for both T2 and T1 imaging. Both the upper and lower thresholds for determining what constitutes lesion were chosen to be two standard deviations from the mean that is present in normal mice. Segmentation of the brain and definition of lesion volumes was performed with a MATLAB (MathWorks®, Natick, MA) program written in-house.

### Histology

Mice were euthanized after final imaging with their brains collected. Hematoxylin and eosin (H&E) sections were generated for each mouse brain and evaluated with an Evos XL (Life Technologies, Carlsbad, CA) digital inverted microscope. Histological slices were graded on a 0–3 scale where 0 represents no lesion and 3 represents a severe lesion as we have previously performed in this model [3, 4]. All of the sections were graded at the same time in a blinded fashion so as to minimize bias.

### Statistics

All analysis testing for statistical significance was performed in SPSS® Statistics (IBM, Armonk, NY). A linear mixed model was used for T2 and T1 lesion volumes while an ordinal regression model was used for the histological grades to test for statistical significance as a function of time (number of weeks post-irradiation), sex, and strain/substrain. In the case of the MRI data, the model controls for repeated measurements and we additionally performed Sidak adjusted pairwise comparisons. All SPSS output files are included in the supplementary data.

## Results

The goal of this study was to determine the effect mouse strain and sex in reference to our previously established model of RN. We analyzed all mice from 4 to 8 weeks post-irradiation as we had previously shown that the former is the onset of lesion and the latter is the peak lesion size on BALB/cN females [3, 4]. We evaluated the induction of radiation necrosis using three metrics: T1-weighted MRI, T2-weighted MRI and a histology score. We had a total of 95 mice where we varied the mouse strain (including substrain), and sex which were evaluated at multiple time points (4, 6 and 8 weeks for MRI and 4 and 8 weeks for histology).

### In general, time post-irradiation and mouse strain significantly affect radiation necrosis modeling, while the effect of sex is minimal

Given the number of variables, we decided to use regression analyses to determine the effect strain, sex and time after irradiation had on the development of radiation necrosis. We evaluated each outcome metric independently. Since the MRI measurements are continuous and appear normally-distributed, we used a multi-linear regression. However, the histology score is an ordinal measurement, so an ordinal model was used in that case. The results of the fixed effects tests are shown in Table 1 for all three outcome variables. Time after radiation, as expected, was found to be a significant factor for all three outcomes (*P* ≤ 0.002). Mouse strain was significant on T2-weighted MRI (*P* = 0.000) but not T1-weighted MRI or histology. However, the interaction term between strain and time was significant for all three measurements (*P* ≤ 0.038) suggesting that strain may be playing a role in radiation necrosis development. Sex by itself was not significant for any measurement (*P* ≥ 0.446) with only the sex and strain interaction term being significant and only for T1-weighted MRI (*P* = .008).

**Table 1:** Type III tests of fixed effects results for T2- and T1-weighted MRI lesion volumes from a linear mixed model and for histological grade from an ordinal model

	*T2-Weighted MRI*			*T1-Weighted MRI*			*Histology*		
Parameter	df	F	Sig.	df	F	Sig.	df	χ^2^	Sig.
Strain	3	16.194	0.000	3	1.388	0.252	3	2.206	0.531
Sex	1	0.005	0.945	1	0.586	0.446	1	0.038	0.845
Time	2	15.015	0.000	2	6.569	0.002	1	32.380	0.000
Sex * Week	2	0.358	0.700	2	0.003	0.997	1	1.193	0.275
Strain * Sex	3	0.668	0.574	3	4.150	0.008	3	3.816	0.282
Strain * Time	6	2.310	0.038	6	2.751	0.015	3	10.650	0.014

The parameters tested (with * indicating interaction between two parameters), parameter degrees of freedom (df), F statistics (F) for the linear model or Wald Chi-square (χ^2^) for the ordinal model, and *P* values (Sig.) are all included. *P* values are Sidak adjusted for MRI data

### There are differences between C57BL/6 and BALB/c mice but not between the N and J substrains of each strain

Within each model, we further performed pairwise comparisons for the MRI data to identify further differences nested within each dependent variable. The statistical results of the pairwise comparisons are shown in Table 2. When looking within the effect of time after irradiation, pooling all the strains and both sexes, week 4 after irradiation is consistently significant versus weeks 6 and 8 (*P* ≤ 0.016). However, there is no statistical significance between week 6 and 8 post-irradiation (*P* ≥ 0.962). When looking within the effect of mouse strain, pooling all the time points and both sexes, we find significant differences between C57BL/6 and BALB/c mice (*P* ≤ 0.015) but not between the N and J substrains within each strain (*P* ≥ 0.145) but only for T2-weighted imaging. Lesion sizes in BALB/c mice are consistently larger than C57BL/6 mice in T2-weighted imaging but not in T1-weighted imaging. For sex, there was no difference between male and female mice. Both findings would be expected from Table 1.

**Table 2:** Pairwise comparisons of the main effects for T2- and T1-weighted MRI lesion volumes from a linear mixed model

		*T2-Weighted MRI*			*T1-Weighted MRI*		
Strain		Mean Diff.	Std. Err.	Sig.	Mean Diff.	Std. Err.	Sig.
BALB/cN	BALB/cJ	7.927	3.508	0.145	4.875	5.321	0.932
	C57BL/6 N	22.757	3.633	0.000	10.065	5.518	0.355
	C57BL/6 J	18.585	3.771	0.000	1.759	5.684	1.000
BALB/cJ	BALB/cN	−7.927	3.508	0.145	−4.875	5.321	0.932
	C57BL/6 N	14.831	3.269	0.000	5.190	4.820	0.867
	C57BL/6 J	10.658	3.417	0.015	−3.117	5.010	0.990
C57BL/6 N	BALB/cN	− 22.757	3.633	0.000	−10.065	5.518	0.355
	BALB/cJ	−14.831	3.269	0.000	−5.190	4.820	0.867
	C57BL/6 J	−4.172	3.511	0.804	−8.306	5.155	0.508
C57BL/6 J	BALB/cJ	−18.585	3.771	0.000	−1.759	5.684	1.000
	BALB/cN	−10.658	3.417	0.015	3.117	5.010	0.990
	C57BL/6 N	4.172	3.511	0.804	8.306	5.155	0.508
		*T2-Weighted MRI*			*T1-Weighted MRI*		
Sex		Mean Diff.	Std. Err.	Sig.	Mean Diff.	Std. Err.	Sig.
F	M	0.178	2.560	0.945	2.926	3.823	0.446
M	F	−0.178	2.560	0.945	−2.926	3.823	0.446
		*T2-Weighted MRI*			*T1-Weighted MRI*		
Time		Mean Diff.	Std. Err.	Sig.	Mean Diff.	Std. Err.	Sig.
4	6	−10.513	2.400	0.000	−12.775	4.046	0.006
	8	−11.610	2.401	0.000	−10.844	3.847	0.016
6	4	10.513	2.400	0.000	12.775	4.046	0.006
	8	−1.097	2.579	0.965	1.931	4.439	0.962
8	4	11.610	2.401	0.000	10.844	3.847	0.016
	6	1.097	2.579	0.965	−1.931	4.439	0.962

The parameters tested, mean differences (Mean Diff.), standard errors (Std. Err.), and *P* values (Sig.) are all included. *P* values are Sidak adjusted

**Fig. 1:**
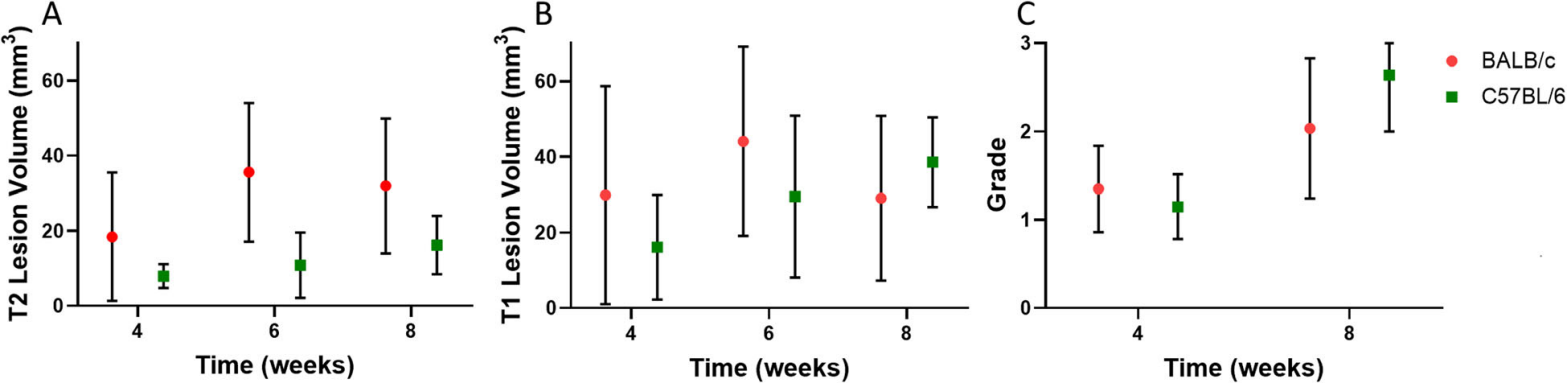
MRI lesion volumes and histological grade as a function of mouse strain and time post-irradiation. Plots show the T2-weighted (Panel **a**) and T1-weighted (Panel **b**) lesion size (in mm^3^) and histological grade (Panel **c**) over time (in weeks) for mice that received 80 Gy. Data includes both substrains for each strain and both sexes. Symbols indicate the mean and the error bars are standard deviation. For T2-weighted scans, BALB/c mice develop radiation necrosis faster and develop larger lesion volumes. T1-weighted scans may indicate a similar result as the T2-weighted scans, although the larger error bars due to more variance in the data make this data harder to interpret. The histological data are harder to interpret, although they do appear to indicate not too much difference on a strain basis

Table 1 noted an interaction effect between mouse strain and time. Statistics for the pairwise comparison for the interaction terms for the MRI data are presented on Table 3. Considering that sex and substrain were not found to be significantly different, Fig. 1 compares the BALB/c strain versus the C57BL/6 strain for all three measurements as a function of time with substrains and sexes averaged out. Again, differences between the strains can be observed on T2 at all time points. The data for T1-weighted lesion volume have a larger variance and thus are harder to interpret, but the data suggests that BALB/c mice might have slightly larger lesions at earlier time points. In fact, we found significant differences between BALB/cN mice and C57BL/6 N at week 4 (*P* = 0.005). The histological data if anything suggest that there might not be large differences at either time point.

**Table 3:** Pairwise comparisons of the interaction effects for T2- and T1-weighted MRI lesion volumes from a linear mixed model

			*T2-Weighted MRI*			*T1-Weighted MRI*		
Sex * Time			Mean Diff.	Std. Err.	Sig.	Mean Diff.	Std. Err.	Sig.
4	F	M	2.169	2.891	0.454	3.059	4.539	0.501
6			0.289	4.626	0.950	2.498	7.526	0.740
8			−1.925	4.006	0.632	3.220	6.327	0.611
Strain * Sex			Mean Diff.	Std. Err	Sig.	Mean Diff.	Std. Err	Sig.
BALB/cN	F	M	5.547	5.113	0.280	19.113	7.993	0.018
BALB/cJ			−0.196	4.363	0.964	0.011	6.433	0.999
C57BL/6 N			−3.716	4.631	0.424	−15.406	6.867	0.027
C57BL/6 J			−0.923	4.881	0.850	7.985	7.195	0.270
Strain * Time			Mean Diff.	Std. Err	Sig.	Mean Diff.	Std. Err	Sig.
4	BALB/cN	BALB/cJ	9.996	3.881	0.064	8.011	6.089	0.718
		C57BL/6 N	15.480	4.088	0.001	21.766	6.419	0.005
		C57BL/6 J	14.568	4.088	0.003	11.819	6.419	0.342
	BALB/cJ	BALB/cN	−9.996	3.881	0.064	−8.011	6.089	0.718
		C57BL/6 N	5.484	4.085	0.699	13.755	6.412	0.184
		C57BL/6 J	4.573	4.085	0.842	3.809	6.412	0.992
	C57BL/6 N	BALB/cN	−15.480	4.088	0.001	−21.766	6.419	0.005
		BALB/cJ	−5.484	4.085	0.699	−13.755	6.412	0.184
		C57BL/6 J	−0.911	4.277	1.000	−9.946	6.717	0.597
	C57BL/6 J	BALB/cN	−14.568	4.088	0.003	−11.819	6.419	0.342
		BALB/cJ	−4.573	4.085	0.842	−3.809	6.412	0.992
		C57BL/6 N	0.911	4.277	1.000	9.946	6.717	0.597
6	BALB/cN	BALB/cJ	12.640	5.945	0.193	8.393	9.736	0.949
		C57BL/6 N	35.301	6.566	0.000	22.012	10.768	0.231
		C57BL/6 J	28.368	6.584	0.000	9.632	10.767	0.939
	BALB/cJ	BALB/cN	−12.640	5.945	0.193	−8.393	9.736	0.949
		C57BL/6 N	22.661	5.491	0.000	13.620	8.762	0.542
		C57BL/6 J	15.728	5.517	0.029	1.239	8.768	1.000
	C57BL/6 N	BALB/cN	−35.301	6.566	0.000	−22.012	10.768	0.231
		BALB/cJ	−22.661	5.491	0.000	−13.620	8.762	0.542
		C57BL/6 J	−6.934	5.928	0.813	−12.381	9.463	0.723
	C57BL/6 J	BALB/cN	−28.368	6.584	0.000	−9.632	10.767	0.939
		BALB/cJ	−15.728	5.517	0.029	−1.239	8.768	1.000
		C57BL/6 N	6.934	5.928	0.813	12.381	9.463	0.723
8	BALB/cN	BALB/cJ	1.143	5.333	1.000	−1.778	8.505	1.000
		C57BL/6 N	17.490	5.345	0.008	−13.583	8.400	0.495
		C57BL/6 J	12.818	5.896	0.173	−16.175	9.275	0.405
	BALB/cJ	BALB/cN	−1.143	5.333	1.000	1.778	8.505	1.000
		C57BL/6 N	16.347	5.042	0.009	−11.806	8.024	0.604
		C57BL/6 J	11.674	5.588	0.209	−14.398	8.874	0.491
	C57BL/6 N	BALB/cN	−17.490	5.345	0.008	13.583	8.400	0.495
		BALB/cJ	−16.347	5.042	0.009	11.806	8.024	0.604
		C57BL/6 J	−4.673	5.592	0.955	−2.592	8.784	1.000
	C57BL/6 J	BALB/cN	−12.818	5.896	0.173	16.175	9.275	0.405
		BALB/cJ	−11.674	5.588	0.209	14.398	8.874	0.491
		C57BL/6 N	4.673	5.592	0.955	2.592	8.784	1.000

The parameter combinations tested, mean differences (Mean Diff.), and *P* values (Sig.) are all included. *P* values are Sidak adjusted. The first column indicates the variable that was held constant whereas the second and third column indicates the parameters that are being compared

## Discussion

The goal was to determine if mouse strain and sex have an impact when modeling radiation necrosis. The rationale was that these two parameters are known to not be consistent in prior reports of murine models of radiation necrosis. Our prior publications have already shown that radiation delivery parameters (dose, device, and fractionation) can all affect the results [3, 4]. Additional biological factors not included in this report but also of interest would be age at irradiation and species, as rats have also been used to model radiation necrosis [12, 13]. We compared BALB/c and C57BL/6 mice of both sexes that received 80 Gy in a single fraction based on MRI-derived lesion volumes and a histological score.

Our results are mixed, with Table 1 and Table 2 showing that strain is a significant factor for T2-weighted lesion volume but not T1-weighted lesion volume or histological score. As T2 imaging could potentially reflect inflammatory changes, further work could be done to see if the neuroinflammatory responses vary on a strain or substrain basis. There is a potential interaction between strain and time after irradiation for T1-weighted lesion volume and histology which still suggests that strain has a significant effect on these outcomes. Overall, our results suggest that BALB/c mice are likely more sensitive to radiation necrosis that C57BL/6 mice. This is consistent with radiosensitivity data previously reported for whole-body irradiation [7, 8]. When looking within strain, we also had included N and J substrains for each but these were not found to be significant. Similarly, sex did not seem to significantly affect our results.

A potential reason for the difference between T1-weighted and T2-weighted lesion volume is that T2-weighted lesion volume was more consistent, with smaller standard deviations and therefore more statistical power. Statistical power also needs to be considered for the histological grade, since due to its ordinal nature we could not use the more powerful parametric statistical analysis. Additional considerations that impact our results include the choice of radiation dose and the consistency of mouse placement in the irradiator. For the latter, if our irradiation setup had included a more tightly shaped mouse immobilizer and more precise localization of the holder itself, it is likely that our mouse to mouse variation would be smaller. Finally, our results are restricted to modeling radiation necrosis which we have previously shown requires very large radiation doses [4, 14]. We cannot guarantee that this would extend to other models of radiation induced pathology, particularly those generated with lower radiation doses.

## Conclusion

The goal of our study was to test the impact of mouse strain and sex when modeling radiation necrosis. Our results show that mouse strain can impact the sensitivity to radiation in a manner consistent with prior work on whole body irradiation. BALB/c mice were found to generate radiation necrosis sooner and to develop potentially larger radiation necrosis lesions than C57BL/6 mice. Though there might be slight differences between the strains, the results seem similar enough that either strain would be viable for modeling necrosis. Though there are known genetic differences within the substrains for BALB/c and C57BL/6 mice, these substrains were not significantly different in their response. Similarly, sex did not appear to modify the response in our model.

## Supplementary information

**Additional file 1.**

## Data Availability

“The datasets used and/or analyzed during the current study are available from the corresponding author on reasonable request.”

## Abbreviations

MRI: Magnetic resonance imaging
H&E: Hematoxylin and eosin
