# Supplementary material for: Impact of mouse strain and sex when modeling radiation necrosis
Source: Radiat Oncol. 2020 Jun 3;15:141. doi: 10.1186/s13014-020-01585-5 (PMC7268332; doi:10.1186/s13014-020-01585-5)
Supplement: Supplementary file 1 — Additional file 1. [file 13014_2020_1585_MOESM1_ESM.pdf]

```
MIXED T2 BY Week Substrain Sex
  /CRITERIA=DFMETHOD(SATTERTHWAITE) CIN(95) MXITER(100) MXSTEP(10) SCORING(1)
    SINGULAR(0.000000000001) HCONVERGE(0, ABSOLUTE) LCONVERGE(0, ABSOLUTE)
PCONVERGE(0.000001, ABSOLUTE)
  /FIXED=Week Substrain Sex Substrain*Week Substrain*Sex Sex*Week | SSTYPE(3)
  /METHOD=REML
  /REPEATED=Week | SUBJECT(Number) COVTYPE(AR1)
  /EMMEANS=TABLES(Substrain) COMPARE ADJ(SIDAK)
  /EMMEANS=TABLES(Sex) COMPARE ADJ(SIDAK)
  /EMMEANS=TABLES(Week) COMPARE ADJ(SIDAK)
  /EMMEANS=TABLES(Sex*Week) COMPARE(Sex) ADJ(SIDAK)
  /EMMEANS=TABLES(Sex*Week) COMPARE(Week) ADJ(SIDAK)
  /EMMEANS=TABLES(Substrain*Sex) COMPARE(Substrain) ADJ(SIDAK)
  /EMMEANS=TABLES(Substrain*Sex) COMPARE(Sex) ADJ(SIDAK)
  /EMMEANS=TABLES(Substrain*Week) COMPARE(Substrain) ADJ(SIDAK)
  /EMMEANS=TABLES(Substrain*Week) COMPARE(Week) ADJ(SIDAK).
```

Mixed Model Analysis

Notes

Output Created		17-JAN-2020 11:59:01
Comments		
Input	Data	
	Active Dataset	DataSet1
	Filter	<none>
	Weight	<none>
	Split File	<none>
	N of Rows in Working Data File	188
Missing Value Handling	Definition of Missing	User-defined missing values are treated as missing.
	Cases Used	Statistics are based on all cases with valid data for all variables in the model.

Syntax	MIXED T2 BY Week<br>Substrain Sex<br><br>/CRITERIA=DFMETHOD(SA<br>TTERTHWAITE) CIN(95)<br>MXITER(100) MXSTEP(10)<br>SCORING(1)<br><br>SINGULAR(0.000000000001<br>) HCONVERGE(0,<br>ABSOLUTE)<br>LCONVERGE(0,<br>ABSOLUTE)<br>PCONVERGE(0.000001,<br>ABSOLUTE)<br>/FIXED=Week Substrain<br>Sex Substrain*Week<br>Substrain*Sex Sex*Week | SSTYPE(3)<br>/METHOD=REML<br>/REPEATED=Week | SUBJECT(Number)<br>COVTYPE(AR1)<br><br>/EMMEANS=TABLES(Substr<br>ain) COMPARE ADJ(SIDAK)<br>/EMMEANS=TABLES(Sex)<br>COMPARE ADJ(SIDAK)<br><br>/EMMEANS=TABLES(Week)<br>COMPARE ADJ(SIDAK)<br><br>/EMMEANS=TABLES(Sex*W<br>eek) COMPARE(Sex)<br>ADJ(SIDAK)<br><br>/EMMEANS=TABLES(Sex*W<br>eek) COMPARE(Week)<br>ADJ(SIDAK)<br><br>/EMMEANS=TABLES(Substr<br>ain*Sex)<br>COMPARE(Substrain)<br>ADJ(SIDAK)

Resources	Processor Time	00:00:00.08
	Elapsed Time	00:00:00.08

Model Dimension[a]

		Number of Levels	Covariance Structure	Number of Parameters	Subject Variables	
Fixed Effects	Intercept	1		1		
	Week	3		2		
	Substrain	4		3		
	Sex	2		1		
	Week * Substrain	12		6		
	Substrain * Sex	8		3		
	Week * Sex	6		2		
Repeated Effects	Week	3	First-Order Autoregressive	2	Number	
Total		39		20		

Information Criteria[a]

-2 Restricted Log Likelihood	1407.882
Akaike's Information Criterion (AIC)	1411.882
Hurvich and Tsai's Criterion (AICC)	1411.954
Bozdogan's Criterion (CAIC)	1420.153
Schwarz's Bayesian Criterion (BIC)	1418.153

The information criteria are displayed in smaller-is-better form.[a]

a. Dependent Variable: T2.

Fixed Effects

Type III Tests of Fixed Effects[a]

Source	Numerator df	Denominator df	F	Sig.
Intercept	1	96.785	259.761	.000
Week	2	123.707	15.015	.000
Substrain	3	94.013	16.194	.000
Sex	1	99.763	.005	.945
Week * Substrain	6	123.433	2.310	.038
Substrain * Sex	3	102.513	.668	.574
Week * Sex	2	125.381	.358	.700

a. Dependent Variable: T2.

Covariance Parameters

Estimates of Covariance Parameters[a]

Parameter		Estimate	Std. Error
Repeated Measures	AR1 diagonal	182.969888	20.396748
	AR1 rho	.250130	.097780

a. Dependent Variable: T2.

Estimated Marginal Means

1. Substrain

Estimates[a]

Substrain	Mean	Std. Error	df	95% Confidence Interval Lower Bound	95% Confidence Interval Upper Bound
1BALB/cN	32.617	2.753	142.462	27.175	38.060
2BALB/cJ	24.691	2.246	76.852	20.219	29.163
3C57BL/6N	9.860	2.385	88.947	5.121	14.600
4C57BL/6J	14.033	2.576	78.910	8.905	19.160

a. Dependent Variable: T2.

Pairwise Comparisons[a]

(I) Substrain	(J) Substrain	Mean Difference (I-J)	Std. Error	df	Sig.[c]	95% Confidence Interval for Difference[c] Lower Bound	
1BALB/cN	2BALB/cJ	7.927	3.508	113.462	.145	-1.468	
	3C57BL/6N	22.757*	3.633	120.718	.000	13.039	
	4C57BL/6J	18.585*	3.771	110.276	.000	8.483	
2BALB/cJ	1BALB/cN	-7.927	3.508	113.462	.145	-17.321	
	3C57BL/6N	14.831*	3.269	83.479	.000	6.022	
	4C57BL/6J	10.658*	3.417	78.014	.015	1.433	
3C57BL/6N	1BALB/cN	-22.757*	3.633	120.718	.000	-32.475	
	2BALB/cJ	-14.831*	3.269	83.479	.000	-23.639	
	4C57BL/6J	-4.172	3.511	83.392	.804	-13.634	
4C57BL/6J	1BALB/cN	-18.585*	3.771	110.276	.000	-28.686	
	2BALB/cJ	-10.658*	3.417	78.014	.015	-19.883	
	3C57BL/6N	4.172	3.511	83.392	.804	-5.289	

Univariate Tests[a]

Numerator df	Denominator df	F	Sig.
3	93.147	16.194	.000

The F tests the effect of Substrain. This test is based on the linearly independent pairwise comparisons among the estimated marginal means.[a]

a. Dependent Variable: T2.

2. Sex

Estimates[a]

Sex	Mean	Std. Error	df	95% Confidence Interval Lower Bound	95% Confidence Interval Upper Bound
F	20.389	1.525	81.465	17.355	23.423
M	20.211	2.031	109.014	16.187	24.236

a. Dependent Variable: T2.

Pairwise Comparisons[a]

(I) Sex	(J) Sex	Mean Difference (I-J)	Std. Error	df	Sig.[b]	95% Confidence Interval for Difference[b] Lower Bound	95% Confidence Interval for Difference[b] Upper Bound
F	M	.178	2.560	99.763	.945	-4.901	5.256
M	F	-.178	2.560	99.763	.945	-5.256	4.901

Based on estimated marginal means[a]

a. Dependent Variable: T2.

b. Adjustment for multiple comparisons: Sidak.

Univariate Tests[a]

Numerator df	Denominator df	F	Sig.
1	99.763	.005	.945

The F tests the effect of Sex. This test is based on the linearly independent pairwise comparisons among the estimated marginal means.[a]

a. Dependent Variable: T2.

3. Week

Estimates[a]

Week	Mean	Std. Error	df	95% Confidence Interval Lower Bound	Upper Bound
4	12.926	1.445	161.007	10.071	15.781
6	23.439	2.185	161.212	19.124	27.753
8	24.536	1.989	163.072	20.609	28.463

a. Dependent Variable: T2.

Pairwise Comparisons[a]

(I) Week	(J) Week	Mean Difference (I-J)	Std. Error	df	Sig.[c]	95% Confidence Interval for Difference[c] Lower Bound	
4	6	-10.513*	2.400	105.783	.000	-16.335	
	8	-11.610*	2.401	168.637	.000	-17.401	
6	4	10.513*	2.400	105.783	.000	4.691	
	8	-1.097	2.579	82.730	.965	-7.382	
8	4	11.610*	2.401	168.637	.000	5.820	
	6	1.097	2.579	82.730	.965	-5.187	

Univariate Tests[a]

Numerator df	Denominator df	F	Sig.
2	109.671	15.015	.000

The F tests the effect of Week. This test is based on the linearly independent pairwise comparisons among the estimated marginal means.[a]

a. Dependent Variable: T2.

4. Sex * Week

Estimates[a]

Sex	Week	Mean	Std. Error	df	95% Confidence Interval	
					Lower Bound	Upper Bound
F	4	14.011	1.945	161.086	10.169	17.852
	6	23.584	2.537	169.751	18.576	28.591
	8	23.574	2.357	161.951	18.919	28.228
M	4	11.841	2.139	160.941	7.618	16.065
	6	23.294	3.717	149.786	15.951	30.638
	8	25.499	3.221	163.504	19.138	31.860

a. Dependent Variable: T2.

Pairwise Comparisons[a]

Week	(I) Sex	(J) Sex	Mean Difference (I-J)	Std. Error	df	Sig.[b]	95% Confidence Interval for Difference[b]	
							Lower Bound	
4	F	M	2.169	2.891	161.007	.454	-3.540	
	M	F	-2.169	2.891	161.007	.454	-7.878	
6	F	M	.289	4.626	156.901	.950	-8.848	
	M	F	-.289	4.626	156.901	.950	-9.427	
8	F	M	-1.925	4.006	162.887	.632	-9.835	
	M	F	1.925	4.006	162.887	.632	-5.986	

Univariate Tests[a]

Week	Numerator df	Denominator df	F	Sig.
4	1	161.007	.563	.454
6	1	156.901	.004	.950
8	1	162.887	.231	.632

Each F tests the simple effects of Sex within each level combination of the other effects shown. These tests are based on the linearly independent pairwise comparisons among the estimated marginal means.[a]

a. Dependent Variable: T2.

5. Sex * Week

Estimates[a]

Sex	Week	Mean	Std. Error	df	95% Confidence Interval Lower Bound	95% Confidence Interval Upper Bound
F	4	14.011	1.945	161.086	10.169	17.852
	6	23.584	2.537	169.751	18.576	28.591
	8	23.574	2.357	161.951	18.919	28.228
M	4	11.841	2.139	160.941	7.618	16.065
	6	23.294	3.717	149.786	15.951	30.638
	8	25.499	3.221	163.504	19.138	31.860

a. Dependent Variable: T2.

Pairwise Comparisons[a]

Sex	(I) Week	(J) Week	Mean Difference (I-J)	Std. Error	df	Sig.[c]	95% Confidence Interval for Difference[c] Lower Bound	

F	4	6	-9.573*	2.878	106.464	.004	-16.554	
		8	-9.563*	2.980	168.315	.005	-16.750	
	6	4	9.573*	2.878	106.464	.004	2.592	
		8	.010	3.038	88.227	1.000	-7.385	
	8	4	9.563*	2.980	168.315	.005	2.377	
		6	-.010	3.038	88.227	1.000	-7.405	
M	4	6	-11.453*	4.012	104.288	.016	-21.190	
		8	-13.657*	3.792	168.997	.001	-22.802	
	6	4	11.453*	4.012	104.288	.016	1.716	
		8	-2.204	4.325	80.887	.941	-12.749	
	8	4	13.657*	3.792	168.997	.001	4.512	
		6	2.204	4.325	80.887	.941	-8.341	

Univariate Tests[a]

Sex	Numerator df	Denominator df	F	Sig.
F	2	110.784	7.309	.001
M	2	113.876	7.740	.001

Each F tests the simple effects of Week within each level combination of the other effects shown. These tests are based on the linearly independent pairwise comparisons among the estimated marginal means.[a]

a. Dependent Variable: T2.

6. Substrain * Sex

Estimates[a]

					95% Confidence Interval	
Substrain	Sex	Mean	Std. Error	df	Lower Bound	Upper Bound
1BALB/cN	F	35.391	2.678	71.735	30.051	40.731
	M	29.844	4.589	167.096	20.784	38.904
2BALB/cJ	F	24.593	2.668	72.627	19.276	29.910
	M	24.789	3.534	82.725	17.760	31.818

3C57BL/6N	F	8.002	3.090	103.063	1.875	14.130
	M	11.718	3.543	82.310	4.670	18.767
4C57BL/6J	F	13.571	3.549	82.052	6.512	20.630
	M	14.494	3.549	82.052	7.435	21.554

a. Dependent Variable: T2.

Pairwise Comparisons[a]

Sex	(I) Substrain	(J) Substrain	Mean Difference (I-J)	Std. Error	df	Sig.[c]	95% Confidence Interval for Difference[c] Lower Bound	
F	1BALB/cN	2BALB/cJ	10.798*	3.776	72.261	.033	.585	
		3C57BL/6N	27.389*	4.089	88.283	.000	16.383	
		4C57BL/6J	21.820*	4.437	78.299	.000	9.844	
	2BALB/cJ	1BALB/cN	-10.798*	3.776	72.261	.033	-21.011	
		3C57BL/6N	16.591*	4.063	89.693	.001	5.659	
		4C57BL/6J	11.022	4.414	79.424	.084	-.888	
	3C57BL/6N	1BALB/cN	-27.389*	4.089	88.283	.000	-38.394	
		2BALB/cJ	-16.591*	4.063	89.693	.001	-27.522	
		4C57BL/6J	-5.569	4.675	91.340	.802	-18.140	
	4C57BL/6J	1BALB/cN	-21.820*	4.437	78.299	.000	-33.796	
		2BALB/cJ	-11.022	4.414	79.424	.084	-22.932	
		3C57BL/6N	5.569	4.675	91.340	.802	-7.002	
M	1BALB/cN	2BALB/cJ	5.055	5.604	142.302	.937	-9.897	
		3C57BL/6N	18.126*	5.648	141.650	.010	3.054	
		4C57BL/6J	15.349*	5.656	140.846	.044	.256	
	2BALB/cJ	1BALB/cN	-5.055	5.604	142.302	.937	-20.007	
		3C57BL/6N	13.070	4.939	84.415	.057	-.236	
		4C57BL/6J	10.294	4.946	84.099	.220	-3.033	
	3C57BL/6N	1BALB/cN	-18.126*	5.648	141.650	.010	-33.197	
		2BALB/cJ	-13.070	4.939	84.415	.057	-26.377	
		4C57BL/6J	-2.776	4.964	83.525	.994	-16.154	
	4C57BL/6J	1BALB/cN	-15.349*	5.656	140.846	.044	-30.442	
		2BALB/cJ	-10.294	4.946	84.099	.220	-23.622	
		3C57BL/6N	2.776	4.964	83.525	.994	-10.602	

Univariate Tests[a]

Sex	Numerator df	Denominator df	F	Sig.
F	3	82.263	17.333	.000
M	3	99.648	4.873	.003

Each F tests the simple effects of Substrain within each level combination of the other effects shown. These tests are based on the linearly independent pairwise comparisons among the estimated marginal means.[a]

a. Dependent Variable: T2.

7. Substrain * Sex

Estimates[a]

					95% Confidence Interval	
Substrain	Sex	Mean	Std. Error	df	Lower Bound	Upper Bound
1BALB/cN	F	35.391	2.678	71.735	30.051	40.731
	M	29.844	4.589	167.096	20.784	38.904
2BALB/cJ	F	24.593	2.668	72.627	19.276	29.910
	M	24.789	3.534	82.725	17.760	31.818
3C57BL/6N	F	8.002	3.090	103.063	1.875	14.130
	M	11.718	3.543	82.310	4.670	18.767
4C57BL/6J	F	13.571	3.549	82.052	6.512	20.630
	M	14.494	3.549	82.052	7.435	21.554

a. Dependent Variable: T2.

Pairwise Comparisons[a]

Substrain	(I) Sex	(J) Sex	Mean Difference (I-J)	Std. Error	df	Sig.[b]	95% Confidence Interval for Difference[b] Lower Bound	
1BALB/cN	F	M	5.547	5.113	151.942	.280	-4.554	
	M	F	-5.547	5.113	151.942	.280	-15.648	
2BALB/cJ	F	M	-.196	4.363	81.123	.964	-8.877	
	M	F	.196	4.363	81.123	.964	-8.485	
3C57BL/6N	F	M	-3.716	4.631	92.711	.424	-12.913	
	M	F	3.716	4.631	92.711	.424	-5.481	
4C57BL/6J	F	M	-.923	4.881	85.705	.850	-10.627	
	M	F	.923	4.881	85.705	.850	-8.781	

Univariate Tests[a]

Substrain	Numerator df	Denominator df	F	Sig.
1BALB/cN	1	151.942	1.177	.280
2BALB/cJ	1	81.123	.002	.964
3C57BL/6N	1	92.711	.644	.424
4C57BL/6J	1	85.705	.036	.850

Each F tests the simple effects of Sex within each level combination of the other effects shown. These tests are based on the linearly independent pairwise comparisons among the estimated marginal means.[a]

a. Dependent Variable: T2.

8. Substrain * Week

Estimates[a]

Substrain	Week	Mean	Std. Error	df	95% Confidence Interval Lower Bound	95% Confidence Interval Upper Bound
1BALB/cN	4	22.937	2.750	160.555	17.506	28.368
	6	42.516	5.062	128.023	32.501	52.532

	8	32.399	4.062	164.535	24.378	40.420
2BALB/cJ	4	12.941	2.746	160.038	7.519	18.364
	6	29.876	3.566	169.643	22.837	36.915
	8	31.256	3.601	161.870	24.145	38.366
3C57BL/6N	4	7.457	3.024	161.520	1.486	13.429
	6	7.215	4.173	167.636	-1.024	15.454
	8	14.909	3.607	161.055	7.785	22.032
4C57BL/6J	4	8.369	3.025	160.941	2.395	14.342
	6	14.148	4.210	169.997	5.838	22.459
	8	19.581	4.273	162.887	11.143	28.020

a. Dependent Variable: T2.

Pairwise Comparisons[a]

Week	(I) Substrain	(J) Substrain	Mean Difference (I-J)	Std. Error	df	Sig.[c]	95% Confidence Interval for Difference[c] Lower Bound	Upper Bound
4	1BALB/cN	2BALB/cJ	9.996	3.881	160.153	.064	-.342	20.333
		3C57BL/6N	15.480*	4.088	160.972	.001	4.590	26.369
		4C57BL/6J	14.568*	4.088	160.767	.003	3.679	25.458
	2BALB/cJ	1BALB/cN	-9.996	3.881	160.153	.064	-20.333	.342
		3C57BL/6N	5.484	4.085	160.719	.699	-5.398	16.366
		4C57BL/6J	4.573	4.085	160.537	.842	-6.309	15.455
	3C57BL/6N	1BALB/cN	-15.480*	4.088	160.972	.001	-26.369	-4.590
		2BALB/cJ	-5.484	4.085	160.719	.699	-16.366	5.398
		4C57BL/6J	-.911	4.277	161.232	1.000	-12.304	10.481
	4C57BL/6J	1BALB/cN	-14.568*	4.088	160.767	.003	-25.458	-3.679
		2BALB/cJ	-4.573	4.085	160.537	.842	-15.455	6.309
		3C57BL/6N	.911	4.277	161.232	1.000	-10.481	12.304
6	1BALB/cN	2BALB/cJ	12.640	5.945	152.258	.193	-3.207	28.488
		3C57BL/6N	35.301*	6.566	147.302	.000	17.791	52.812
		4C57BL/6J	28.368*	6.584	152.696	.000	10.818	45.917
	2BALB/cJ	1BALB/cN	-12.640	5.945	152.258	.193	-28.488	3.207
		3C57BL/6N	22.661*	5.491	169.541	.000	8.044	37.278
		4C57BL/6J	15.728*	5.517	169.919	.029	1.041	30.414
	3C57BL/6N	1BALB/cN	-35.301*	6.566	147.302	.000	-52.812	-17.791
		2BALB/cJ	-22.661*	5.491	169.541	.000	-37.278	-8.044

		4C57BL/6J	-6.934	5.928	169.457	.813	-22.714	8.847
	4C57BL/6J	1BALB/cN	-28.368*	6.584	152.696	.000	-45.917	-10.818
		2BALB/cJ	-15.728*	5.517	169.919	.029	-30.414	-1.041
		3C57BL/6N	6.934	5.928	169.457	.813	-8.847	22.714
8	1BALB/cN	2BALB/cJ	1.143	5.333	163.544	1.000	-13.061	15.348
		3C57BL/6N	17.490*	5.345	162.846	.008	3.254	31.727
		4C57BL/6J	12.818	5.896	163.693	.173	-2.885	28.520
	2BALB/cJ	1BALB/cN	-1.143	5.333	163.544	1.000	-15.348	13.061
		3C57BL/6N	16.347*	5.042	160.812	.009	2.916	29.777
		4C57BL/6J	11.674	5.588	162.472	.209	-3.210	26.558
	3C57BL/6N	1BALB/cN	-17.490*	5.345	162.846	.008	-31.727	-3.254
		2BALB/cJ	-16.347*	5.042	160.812	.009	-29.777	-2.916
		4C57BL/6J	-4.673	5.592	162.146	.955	-19.568	10.223
	4C57BL/6J	1BALB/cN	-12.818	5.896	163.693	.173	-28.520	2.885
		2BALB/cJ	-11.674	5.588	162.472	.209	-26.558	3.210
		3C57BL/6N	4.673	5.592	162.146	.955	-10.223	19.568

Based on estimated marginal means[a]

*. The mean difference is significant at the .05 level.

a. Dependent Variable: T2.

c. Adjustment for multiple comparisons: Sidak.

Univariate Tests[a]

Week	Numerator df	Denominator df	F	Sig.
4	3	160.711	6.228	.001
6	3	157.984	11.978	.000
8	3	162.490	5.332	.002

Each F tests the simple effects of Substrain within each level combination of the other effects shown. These tests are based on the linearly independent pairwise comparisons among the estimated marginal means.[a]

a. Dependent Variable: T2.

9. Substrain * Week

Estimates[a]

Substrain	Week	Mean	Std. Error	df	95% Confidence Interval Lower Bound	95% Confidence Interval Upper Bound
1BALB/cN	4	22.937	2.750	160.555	17.506	28.368
	6	42.516	5.062	128.023	32.501	52.532
	8	32.399	4.062	164.535	24.378	40.420
2BALB/cJ	4	12.941	2.746	160.038	7.519	18.364
	6	29.876	3.566	169.643	22.837	36.915
	8	31.256	3.601	161.870	24.145	38.366
3C57BL/6N	4	7.457	3.024	161.520	1.486	13.429
	6	7.215	4.173	167.636	-1.024	15.454
	8	14.909	3.607	161.055	7.785	22.032
4C57BL/6J	4	8.369	3.025	160.941	2.395	14.342
	6	14.148	4.210	169.997	5.838	22.459
	8	19.581	4.273	162.887	11.143	28.020

a. Dependent Variable: T2.

Pairwise Comparisons[a]

Substrain	(I) Week	(J) Week	Mean Difference (I-J)	Std. Error	df	Sig.[c]	95% Confidence Interval for Difference[c] Lower Bound	
1BALB/cN	4	6	-19.579*	5.293	93.633	.001	-32.447	
		8	-9.462	4.714	168.343	.133	-20.832	
	6	4	19.579*	5.293	93.633	.001	6.711	
		8	10.117	5.531	81.220	.198	-3.366	
	8	4	9.462	4.714	168.343	.133	-1.908	
		6	-10.117	5.531	81.220	.198	-23.601	
2BALB/cJ	4	6	-16.935*	4.024	109.298	.000	-26.692	
		8	-18.314*	4.376	167.309	.000	-28.868	
	6	4	16.935*	4.024	109.298	.000	7.178	
		8	-1.380	4.352	83.127	.985	-11.984	
	8	4	18.314*	4.376	167.309	.000	7.760	

		6	1.380	4.352	83.127	.985	-9.225	
3C57BL/6N	4	6	.242	4.697	108.110	1.000	-11.149	
		8	-7.451	4.620	169.675	.292	-18.592	
	6	4	-.242	4.697	108.110	1.000	-11.634	
		8	-7.694	4.910	92.874	.320	-19.633	
	8	4	7.451	4.620	169.675	.292	-3.689	
		6	7.694	4.910	92.874	.320	-4.246	
4C57BL/6J	4	6	-5.780	4.722	115.430	.532	-17.221	
		8	-11.213	5.125	168.429	.087	-23.573	
	6	4	5.780	4.722	115.430	.532	-5.661	
		8	-5.433	5.208	81.977	.657	-18.126	
	8	4	11.213	5.125	168.429	.087	-1.147	
		6	5.433	5.208	81.977	.657	-7.261	

Univariate Tests[a]

Substrain	Numerator df	Denominator df	F	Sig.
1BALB/cN	2	117.240	7.021	.001
2BALB/cJ	2	103.132	12.018	.000
3C57BL/6N	2	119.745	1.669	.193
4C57BL/6J	2	103.191	2.434	.093

Each F tests the simple effects of Week within each level combination of the other effects shown. These tests are based on the linearly independent pairwise comparisons among the estimated marginal means.[a]

a. Dependent Variable: T2.

```
MIXED T1 BY Week Substrain Sex
  /CRITERIA=DFMETHOD(SATTERTHWAITE) CIN(95) MXITER(100) MXSTEP(10) SCORING(1)
    SINGULAR(0.000000000001) HCONVERGE(0, ABSOLUTE) LCONVERGE(0, ABSOLUTE)
PCONVERGE(0.000001, ABSOLUTE)
  /FIXED=Week Substrain Sex Substrain*Week Substrain*Sex Sex*Week | SSTYPE(3)
  /METHOD=REML
  /REPEATED=Week | SUBJECT(Number) COVTYPE(AR1)
  /EMMEANS=TABLES(Substrain) COMPARE ADJ(SIDAK)
  /EMMEANS=TABLES(Sex) COMPARE ADJ(SIDAK)
  /EMMEANS=TABLES(Week) COMPARE ADJ(SIDAK)
  /EMMEANS=TABLES(Sex*Week) COMPARE(Sex) ADJ(SIDAK)
  /EMMEANS=TABLES(Sex*Week) COMPARE(Week) ADJ(SIDAK)
  /EMMEANS=TABLES(Substrain*Sex) COMPARE(Substrain) ADJ(SIDAK)
  /EMMEANS=TABLES(Substrain*Sex) COMPARE(Sex) ADJ(SIDAK)
  /EMMEANS=TABLES(Substrain*Week) COMPARE(Substrain) ADJ(SIDAK)
  /EMMEANS=TABLES(Substrain*Week) COMPARE(Week) ADJ(SIDAK).
```

Mixed Model Analysis

Notes

Output Created		17-JAN-2020 11:59:48
Comments		
Input	Data	
	Active Dataset	DataSet1
	Filter	<none>
	Weight	<none>
	Split File	<none>
	N of Rows in Working Data File	188
Missing Value Handling	Definition of Missing	User-defined missing values are treated as missing.
	Cases Used	Statistics are based on all cases with valid data for all variables in the model.

Syntax	MIXED T1 BY Week Substrain Sex<br><br>/CRITERIA=DFMETHOD(SATTERTHWAITE) CIN(95) MXITER(100) MXSTEP(10) SCORING(1)<br><br>SINGULAR(0.000000000001) HCONVERGE(0, ABSOLUTE) LCONVERGE(0, ABSOLUTE) PCONVERGE(0.000001, ABSOLUTE)<br>/FIXED=Week Substrain Sex Substrain*Week Substrain*Sex Sex*Week | SSTYPE(3)<br>/METHOD=REML<br>/REPEATED=Week | SUBJECT(Number) COVTYPE(AR1)<br><br>/EMMEANS=TABLES(Substrain) COMPARE ADJ(SIDAK)<br>/EMMEANS=TABLES(Sex) COMPARE ADJ(SIDAK)<br><br>/EMMEANS=TABLES(Week) COMPARE ADJ(SIDAK)<br><br>/EMMEANS=TABLES(Sex*Week) COMPARE(Sex) ADJ(SIDAK)<br><br>/EMMEANS=TABLES(Sex*Week) COMPARE(Week) ADJ(SIDAK)<br><br>/EMMEANS=TABLES(Substrain*Sex) COMPARE(Substrain) ADJ(SIDAK)

Resources	Processor Time	00:00:00.06
	Elapsed Time	00:00:00.05

Model Dimension[a]

		Number of Levels	Covariance Structure	Number of Parameters	Subject Variables	
Fixed Effects	Intercept	1		1		
	Week	3		2		
	Substrain	4		3		
	Sex	2		1		
	Week * Substrain	12		6		
	Substrain * Sex	8		3		
	Week * Sex	6		2		
Repeated Effects	Week	3	First-Order Autoregressive	2	Number	
Total		39		20		

Information Criteria[a]

-2 Restricted Log Likelihood	1556.782
Akaike's Information Criterion (AIC)	1560.782
Hurvich and Tsai's Criterion (AICC)	1560.855
Bozdogan's Criterion (CAIC)	1569.042
Schwarz's Bayesian Criterion (BIC)	1567.042

The information criteria are displayed in smaller-is-better form.[a]

a. Dependent Variable: T1.

Fixed Effects

Type III Tests of Fixed Effects[a]

Source	Numerator df	Denominator df	F	Sig.
Intercept	1	92.973	261.634	.000
Week	2	127.193	6.569	.002
Substrain	3	87.951	1.388	.252
Sex	1	95.086	.586	.446
Week * Substrain	6	128.161	2.751	.015
Substrain * Sex	3	103.115	4.150	.008
Week * Sex	2	128.609	.003	.997

a. Dependent Variable: T1.

Covariance Parameters

Estimates of Covariance Parameters[a]

Parameter		Estimate	Std. Error
Repeated Measures	AR1 diagonal	451.167119	49.398952
	AR1 rho	.108640	.108742

a. Dependent Variable: T1.

Estimated Marginal Means

1. Substrain

Estimates[a]

Substrain	Mean	Std. Error	df	95% Confidence Interval Lower Bound	95% Confidence Interval Upper Bound
1BALB/cN	34.554	4.257	152.813	26.144	42.964
2BALB/cJ	29.679	3.303	69.774	23.091	36.267
3C57BL/6N	24.490	3.519	78.046	17.483	31.496
4C57BL/6J	32.796	3.767	69.363	25.282	40.310

a. Dependent Variable: T1.

Pairwise Comparisons[a]

(I) Substrain	(J) Substrain	Mean Difference (I-J)	Std. Error	df	Sig.[b]	95% Confidence Interval for Difference[b] Lower Bound	
1BALB/cN	2BALB/cJ	4.875	5.321	119.746	.932	-9.361	
	3C57BL/6N	10.065	5.518	123.589	.355	-4.690	
	4C57BL/6J	1.759	5.684	112.480	1.000	-13.465	
2BALB/cJ	1BALB/cN	-4.875	5.321	119.746	.932	-19.111	
	3C57BL/6N	5.190	4.820	74.439	.867	-7.838	
	4C57BL/6J	-3.117	5.010	69.541	.990	-16.683	
3C57BL/6N	1BALB/cN	-10.065	5.518	123.589	.355	-24.819	
	2BALB/cJ	-5.190	4.820	74.439	.867	-18.218	
	4C57BL/6J	-8.306	5.155	73.268	.508	-22.246	
4C57BL/6J	1BALB/cN	-1.759	5.684	112.480	1.000	-16.982	
	2BALB/cJ	3.117	5.010	69.541	.990	-10.450	
	3C57BL/6N	8.306	5.155	73.268	.508	-5.633	

Univariate Tests[a]

Numerator df	Denominator df	F	Sig.
3	86.115	1.388	.252

The F tests the effect of Substrain. This test is based on the linearly independent pairwise comparisons among the estimated marginal means.[a]

a. Dependent Variable: T1.

2. Sex

Estimates[a]

Sex	Mean	Std. Error	df	95% Confidence Interval Lower Bound	95% Confidence Interval Upper Bound
F	31.843	2.243	73.137	27.371	36.314
M	28.917	3.055	107.455	22.861	34.972

a. Dependent Variable: T1.

Pairwise Comparisons[a]

(I) Sex	(J) Sex	Mean Difference (I-J)	Std. Error	df	Sig.[b]	95% Confidence Interval for Difference[b] Lower Bound	95% Confidence Interval for Difference[b] Upper Bound
F	M	2.926	3.823	95.086	.446	-4.665	10.516
M	F	-2.926	3.823	95.086	.446	-10.516	4.665

Based on estimated marginal means[a]

a. Dependent Variable: T1.

b. Adjustment for multiple comparisons: Sidak.

Univariate Tests[a]

Numerator df	Denominator df	F	Sig.
1	95.086	.586	.446

The F tests the effect of Sex. This test is based on the linearly independent pairwise comparisons among the estimated marginal means.[a]

a. Dependent Variable: T1.

3. Week

Estimates[a]

Week	Mean	Std. Error	df	95% Confidence Interval Lower Bound	95% Confidence Interval Upper Bound
4	22.506	2.270	166.753	18.025	26.988
6	35.282	3.542	157.697	28.286	42.278
8	33.351	3.138	166.985	27.156	39.546

a. Dependent Variable: T1.

Pairwise Comparisons[a]

(I) Week	(J) Week	Mean Difference (I-J)	Std. Error	df	Sig.[c]	95% Confidence Interval for Difference[c] Lower Bound	
4	6	-12.775*	4.046	104.729	.006	-22.594	
	8	-10.844*	3.847	168.846	.016	-20.123	
6	4	12.775*	4.046	104.729	.006	2.957	
	8	1.931	4.439	81.275	.962	-8.891	
8	4	10.844*	3.847	168.846	.016	1.565	
	6	-1.931	4.439	81.275	.962	-12.754	

Univariate Tests[a]

Numerator df	Denominator df	F	Sig.
2	114.535	6.569	.002

The F tests the effect of Week. This test is based on the linearly independent pairwise comparisons among the estimated marginal means.[a]

a. Dependent Variable: T1.

4. Sex * Week

Estimates[a]

Sex	Week	Mean	Std. Error	df	95% Confidence Interval Lower Bound	95% Confidence Interval Upper Bound
F	4	24.036	3.054	166.661	18.006	30.066
	6	36.531	4.046	168.916	28.544	44.519
	8	34.961	3.747	166.781	27.563	42.359
M	4	20.977	3.358	166.828	14.347	27.608
	6	34.033	6.086	145.418	22.004	46.062
	8	31.741	5.066	167.048	21.739	41.742

a. Dependent Variable: T1.

Pairwise Comparisons[a]

Week	(I) Sex	(J) Sex	Mean Difference (I-J)	Std. Error	df	Sig.[b]	95% Confidence Interval for Difference[b] Lower Bound
4	F	M	3.059	4.539	166.753	.501	-5.903
	M	F	-3.059	4.539	166.753	.501	-12.021
6	F	M	2.498	7.526	154.841	.740	-12.369
	M	F	-2.498	7.526	154.841	.740	-17.366
8	F	M	3.220	6.327	166.927	.611	-9.270
	M	F	-3.220	6.327	166.927	.611	-15.710

Univariate Tests[a]

Week	Numerator df	Denominator df	F	Sig.
4	1	166.753	.454	.501
6	1	154.841	.110	.740
8	1	166.927	.259	.611

Each F tests the simple effects of Sex within each level combination of the other effects shown. These tests are based on the linearly independent pairwise comparisons among the estimated marginal means.[a]

a. Dependent Variable: T1.

5. Sex * Week

Estimates[a]

Sex	Week	Mean	Std. Error	df	95% Confidence Interval	
					Lower Bound	Upper Bound
F	4	24.036	3.054	166.661	18.006	30.066
	6	36.531	4.046	168.916	28.544	44.519
	8	34.961	3.747	166.781	27.563	42.359
M	4	20.977	3.358	166.828	14.347	27.608
	6	34.033	6.086	145.418	22.004	46.062
	8	31.741	5.066	167.048	21.739	41.742

a. Dependent Variable: T1.

Pairwise Comparisons[a]

Sex	(I) Week	(J) Week	Mean Difference (I-J)	Std. Error	df	Sig.[c]	95% Confidence Interval for Difference[c]	
							Lower Bound	

F	4	6	-12.495*	4.848	106.726	.034	-24.256	
		8	-10.925	4.802	168.792	.071	-22.506	
	6	4	12.495*	4.848	106.726	.034	.735	
		8	1.570	5.230	88.728	.987	-11.158	
	8	4	10.925	4.802	168.792	.071	-.656	
		6	-1.570	5.230	88.728	.987	-14.299	
M	4	6	-13.056	6.773	103.571	.160	-29.493	
		8	-10.764	6.056	168.905	.214	-25.369	
	6	4	13.056	6.773	103.571	.160	-3.382	
		8	2.292	7.445	79.519	.986	-15.867	
	8	4	10.764	6.056	168.905	.214	-3.842	
		6	-2.292	7.445	79.519	.986	-20.451	

Univariate Tests[a]

Sex	Numerator df	Denominator df	F	Sig.
F	2	115.967	4.206	.017
M	2	120.564	2.588	.079

Each F tests the simple effects of Week within each level combination of the other effects shown. These tests are based on the linearly independent pairwise comparisons among the estimated marginal means.[a]

a. Dependent Variable: T1.

6. Substrain * Sex

Estimates[a]

					95% Confidence Interval	
Substrain	Sex	Mean	Std. Error	df	Lower Bound	Upper Bound
1BALB/cN	F	44.111	3.892	64.725	36.337	51.885
	M	24.998	7.283	168.182	10.620	39.375
2BALB/cJ	F	29.685	3.945	68.745	21.814	37.555
	M	29.674	5.191	74.494	19.331	40.016

3C57BL/6N	F	16.786	4.614	92.675	7.623	25.949
	M	32.193	5.202	73.920	21.827	42.559
4C57BL/6J	F	36.788	5.209	73.700	26.409	47.167
	M	28.803	5.209	73.700	18.424	39.182

a. Dependent Variable: T1.

Pairwise Comparisons[a]

Sex	(I) Substrain	(J) Substrain	Mean Difference (I-J)	Std. Error	df	Sig.[c]	95% Confidence Interval for Difference[c] Lower Bound	
F	1BALB/cN	2BALB/cJ	14.426	5.534	66.819	.066	-.578	
		3C57BL/6N	27.325*	6.037	79.754	.000	11.038	
		4C57BL/6J	7.323	6.490	70.527	.840	-10.245	
	2BALB/cJ	1BALB/cN	-14.426	5.534	66.819	.066	-29.431	
		3C57BL/6N	12.898	6.053	83.114	.198	-3.414	
		4C57BL/6J	-7.104	6.502	73.046	.859	-24.686	
	3C57BL/6N	1BALB/cN	-27.325*	6.037	79.754	.000	-43.612	
		2BALB/cJ	-12.898	6.053	83.114	.198	-29.211	
		4C57BL/6J	-20.002*	6.920	83.112	.029	-38.653	
	4C57BL/6J	1BALB/cN	-7.323	6.490	70.527	.840	-24.891	
		2BALB/cJ	7.104	6.502	73.046	.859	-10.478	
		3C57BL/6N	20.002*	6.920	83.112	.029	1.351	
M	1BALB/cN	2BALB/cJ	-4.676	8.681	157.591	.995	-27.807	
		3C57BL/6N	-7.195	8.742	156.249	.959	-30.491	
		4C57BL/6J	-3.806	8.749	155.605	.999	-27.121	
	2BALB/cJ	1BALB/cN	4.676	8.681	157.591	.995	-18.454	
		3C57BL/6N	-2.519	7.270	76.812	1.000	-22.151	
		4C57BL/6J	.870	7.278	76.557	1.000	-18.787	
	3C57BL/6N	1BALB/cN	7.195	8.742	156.249	.959	-16.100	
		2BALB/cJ	2.519	7.270	76.812	1.000	-17.113	
		4C57BL/6J	3.390	7.299	75.715	.998	-16.329	
	4C57BL/6J	1BALB/cN	3.806	8.749	155.605	.999	-19.509	
		2BALB/cJ	-.870	7.278	76.557	1.000	-20.528	
		3C57BL/6N	-3.390	7.299	75.715	.998	-23.108	

Univariate Tests[a]

Sex	Numerator df	Denominator df	F	Sig.
F	3	75.139	7.240	.000
M	3	93.217	.233	.873

Each F tests the simple effects of Substrain within each level combination of the other effects shown. These tests are based on the linearly independent pairwise comparisons among the estimated marginal means.[a]

a. Dependent Variable: T1.

7. Substrain * Sex

Estimates[a]

					95% Confidence Interval	
Substrain	Sex	Mean	Std. Error	df	Lower Bound	Upper Bound
1BALB/cN	F	44.111	3.892	64.725	36.337	51.885
	M	24.998	7.283	168.182	10.620	39.375
2BALB/cJ	F	29.685	3.945	68.745	21.814	37.555
	M	29.674	5.191	74.494	19.331	40.016
3C57BL/6N	F	16.786	4.614	92.675	7.623	25.949
	M	32.193	5.202	73.920	21.827	42.559
4C57BL/6J	F	36.788	5.209	73.700	26.409	47.167
	M	28.803	5.209	73.700	18.424	39.182

a. Dependent Variable: T1.

Pairwise Comparisons[a]

Substrain	(I) Sex	(J) Sex	Mean Difference (I-J)	Std. Error	df	Sig.[c]	95% Confidence Interval for Difference[c] Lower Bound	
1BALB/cN	F	M	19.113*	7.993	166.723	.018	3.333	
	M	F	-19.113*	7.993	166.723	.018	-34.894	
2BALB/cJ	F	M	.011	6.433	75.136	.999	-12.804	
	M	F	-.011	6.433	75.136	.999	-12.827	
3C57BL/6N	F	M	-15.406*	6.867	85.615	.027	-29.059	
	M	F	15.406*	6.867	85.615	.027	1.754	
4C57BL/6J	F	M	7.985	7.195	78.805	.270	-6.336	
	M	F	-7.985	7.195	78.805	.270	-22.306	

Univariate Tests[a]

Substrain	Numerator df	Denominator df	F	Sig.
1BALB/cN	1	166.723	5.718	.018
2BALB/cJ	1	75.136	.000	.999
3C57BL/6N	1	85.615	5.033	.027
4C57BL/6J	1	78.805	1.232	.270

Each F tests the simple effects of Sex within each level combination of the other effects shown. These tests are based on the linearly independent pairwise comparisons among the estimated marginal means.[a]

a. Dependent Variable: T1.

8. Substrain * Week

Estimates[a]

Substrain	Week	Mean	Std. Error	df	95% Confidence Interval Lower Bound	95% Confidence Interval Upper Bound
1BALB/cN	4	32.905	4.317	166.727	24.382	41.429
	6	45.291	8.431	118.628	28.597	61.985

	8	25.466	6.396	167.356	12.839	38.093
2BALB/cJ	4	24.895	4.308	166.320	16.390	33.400
	6	36.898	5.659	168.873	25.728	48.069
	8	27.244	5.799	166.714	15.796	38.693
3C57BL/6N	4	11.140	4.750	166.856	1.763	20.517
	6	23.279	6.686	168.445	10.080	36.477
	8	39.050	5.660	166.039	27.874	50.226
4C57BL/6J	4	21.086	4.750	166.828	11.709	30.463
	6	35.659	6.697	168.996	22.439	48.880
	8	41.642	6.717	166.923	28.381	54.902

a. Dependent Variable: T1.

Pairwise Comparisons[a]

Week	(I) Substrain	(J) Substrain	Mean Difference (I-J)	Std. Error	df	Sig.[c]	95% Confidence Interval for Difference[c] Lower Bound	Upper Bound
4	1BALB/cN	2BALB/cJ	8.011	6.089	166.488	.718	-8.204	24.225
		3C57BL/6N	21.766*	6.419	166.765	.005	4.675	38.856
		4C57BL/6J	11.819	6.419	166.783	.342	-5.271	28.910
	2BALB/cJ	1BALB/cN	-8.011	6.089	166.488	.718	-24.225	8.204
		3C57BL/6N	13.755	6.412	166.586	.184	-3.319	30.829
		4C57BL/6J	3.809	6.412	166.605	.992	-13.265	20.882
	3C57BL/6N	1BALB/cN	-21.766*	6.419	166.765	.005	-38.856	-4.675
		2BALB/cJ	-13.755	6.412	166.586	.184	-30.829	3.319
		4C57BL/6J	-9.946	6.717	166.842	.597	-27.831	7.938
	4C57BL/6J	1BALB/cN	-11.819	6.419	166.783	.342	-28.910	5.271
		2BALB/cJ	-3.809	6.412	166.605	.992	-20.882	13.265
		3C57BL/6N	9.946	6.717	166.842	.597	-7.938	27.831
6	1BALB/cN	2BALB/cJ	8.393	9.736	142.770	.949	-17.583	34.369
		3C57BL/6N	22.012	10.768	144.294	.231	-6.712	50.737
		4C57BL/6J	9.632	10.767	145.926	.939	-19.086	38.349
	2BALB/cJ	1BALB/cN	-8.393	9.736	142.770	.949	-34.369	17.583
		3C57BL/6N	13.620	8.762	168.940	.542	-9.706	36.945
		4C57BL/6J	1.239	8.768	168.966	1.000	-22.102	24.580
	3C57BL/6N	1BALB/cN	-22.012	10.768	144.294	.231	-50.737	6.712
		2BALB/cJ	-13.620	8.762	168.940	.542	-36.945	9.706

		4C57BL/6J	-12.381	9.463	168.884	.723	-37.573	12.812
	4C57BL/6J	1BALB/cN	-9.632	10.767	145.926	.939	-38.349	19.086
		2BALB/cJ	-1.239	8.768	168.966	1.000	-24.580	22.102
		3C57BL/6N	12.381	9.463	168.884	.723	-12.812	37.573
8	1BALB/cN	2BALB/cJ	-1.778	8.505	167.148	1.000	-24.423	20.868
		3C57BL/6N	-13.583	8.400	166.737	.495	-35.950	8.783
		4C57BL/6J	-16.175	9.275	167.135	.405	-40.870	8.519
	2BALB/cJ	1BALB/cN	1.778	8.505	167.148	1.000	-20.868	24.423
		3C57BL/6N	-11.806	8.024	165.967	.604	-33.173	9.561
		4C57BL/6J	-14.398	8.874	166.835	.491	-38.025	9.229
	3C57BL/6N	1BALB/cN	13.583	8.400	166.737	.495	-8.783	35.950
		2BALB/cJ	11.806	8.024	165.967	.604	-9.561	33.173
		4C57BL/6J	-2.592	8.784	166.575	1.000	-25.980	20.796
	4C57BL/6J	1BALB/cN	16.175	9.275	167.135	.405	-8.519	40.870
		2BALB/cJ	14.398	8.874	166.835	.491	-9.229	38.025
		3C57BL/6N	2.592	8.784	166.575	1.000	-20.796	25.980

Based on estimated marginal means[a]

*. The mean difference is significant at the .05 level.

a. Dependent Variable: T1.

c. Adjustment for multiple comparisons: Sidak.

Univariate Tests[a]

Week	Numerator df	Denominator df	F	Sig.
4	3	166.672	3.949	.009
6	3	153.789	1.524	.211
8	3	166.691	1.739	.161

Each F tests the simple effects of Substrain within each level combination of the other effects shown. These tests are based on the linearly independent pairwise comparisons among the estimated marginal means.[a]

a. Dependent Variable: T1.

9. Substrain * Week

Estimates[a]

Substrain	Week	Mean	Std. Error	df	95% Confidence Interval Lower Bound	95% Confidence Interval Upper Bound
1BALB/cN	4	32.905	4.317	166.727	24.382	41.429
	6	45.291	8.431	118.628	28.597	61.985
	8	25.466	6.396	167.356	12.839	38.093
2BALB/cJ	4	24.895	4.308	166.320	16.390	33.400
	6	36.898	5.659	168.873	25.728	48.069
	8	27.244	5.799	166.714	15.796	38.693
3C57BL/6N	4	11.140	4.750	166.856	1.763	20.517
	6	23.279	6.686	168.445	10.080	36.477
	8	39.050	5.660	166.039	27.874	50.226
4C57BL/6J	4	21.086	4.750	166.828	11.709	30.463
	6	35.659	6.697	168.996	22.439	48.880
	8	41.642	6.717	166.923	28.381	54.902

a. Dependent Variable: T1.

Pairwise Comparisons[a]

Substrain	(I) Week	(J) Week	Mean Difference (I-J)	Std. Error	df	Sig.[c]	95% Confidence Interval for Difference[c] Lower Bound	
1BALB/cN	4	6	-12.386	9.013	91.469	.434	-34.308	
		8	7.439	7.540	168.800	.693	-10.745	
	6	4	12.386	9.013	91.469	.434	-9.536	
		8	19.825	9.523	79.184	.117	-3.405	
	8	4	-7.439	7.540	168.800	.693	-25.622	
		6	-19.825	9.523	79.184	.117	-43.054	
2BALB/cJ	4	6	-12.004	6.768	107.866	.219	-28.419	
		8	-2.349	7.134	168.590	.983	-19.555	
	6	4	12.004	6.768	107.866	.219	-4.412	
		8	9.654	7.598	83.260	.502	-8.859	
	8	4	2.349	7.134	168.590	.983	-14.856	

		6	-9.654	7.598	83.260	.502	-28.167	
3C57BL/6N	4	6	-12.139	7.898	110.796	.335	-31.286	
		8	-27.910*	7.363	168.998	.001	-45.668	
	6	4	12.139	7.898	110.796	.335	-7.008	
		8	-15.771	8.356	94.281	.175	-36.083	
	8	4	27.910*	7.363	168.998	.001	10.152	
		6	15.771	8.356	94.281	.175	-4.541	
4C57BL/6J	4	6	-14.574	7.906	114.943	.190	-33.730	
		8	-20.556*	8.194	168.810	.039	-40.317	
	6	4	14.574	7.906	114.943	.190	-4.583	
		8	-5.982	8.957	80.148	.880	-27.824	
	8	4	20.556*	8.194	168.810	.039	.795	
		6	5.982	8.957	80.148	.880	-15.859	

Univariate Tests[a]

Substrain	Numerator df	Denominator df	F	Sig.
1BALB/cN	2	122.798	2.167	.119
2BALB/cJ	2	105.540	1.662	.195
3C57BL/6N	2	126.779	7.184	.001
4C57BL/6J	2	104.932	3.606	.031

Each F tests the simple effects of Week within each level combination of the other effects shown. These tests are based on the linearly independent pairwise comparisons among the estimated marginal means.[a]

a. Dependent Variable: T1.

```
GENLIN Grade (ORDER=DESCENDING) BY Week Substrain Sex (ORDER=DESCENDING)
  /MODEL Week Substrain Sex Substrain*Week Substrain*Sex Sex*Week
 DISTRIBUTION=MULTINOMIAL LINK=CUMLOGIT
  /CRITERIA METHOD=FISHER(1) SCALE=1 COVB=MODEL MAXITERATIONS=100
MAXSTEPHALVING=5
    PCONVERGE=1E-006(ABSOLUTE) SINGULAR=1E-012 ANALYSISTYPE=3(WALD) CILEVEL=95
CITYPE=WALD
    LIKELIHOOD=FULL
  /MISSING CLASSMISSING=EXCLUDE
  /PRINT CPS DESCRIPTIVES MODELINFO FIT SUMMARY SOLUTION.
```

Generalized Linear Models

Notes

Output Created		17-JAN-2020 12:00:23
Comments		
Input	Data	
	Active Dataset	DataSet1
	Filter	<none>
	Weight	<none>
	Split File	<none>
	N of Rows in Working Data File	188
Missing Value Handling	Definition of Missing	User-defined missing values for factor, subject and within-subject variables are treated as missing.
	Cases Used	Statistics are based on cases with valid data for all variables in the model.
Weight Handling		not applicable

Syntax		GENLIN Grade (ORDER=DESCENDING) BY Week Substrain Sex (ORDER=DESCENDING) /MODEL Week Substrain Sex Substrain*Week Substrain*Sex Sex*Week DISTRIBUTION=MULTINOMIAL LINK=CUMLOGIT /CRITERIA METHOD=FISHER(1) SCALE=1 COVB=MODEL MAXITERATIONS=100 MAXSTEPHALVING=5 PCONVERGE=1E-006(ABSOLUTE) SINGULAR=1E-012 ANALYSISTYPE=3(WALD) CILEVEL=95 CITYPE=WALD LIKELIHOOD=FULL /MISSING CLASSMISSING=EXCLUDE /PRINT CPS DESCRIPTIVES MODELINFO FIT SUMMARY SOLUTION.
Resources	Processor Time	00:00:00.05
	Elapsed Time	00:00:00.14

Model Information

Dependent Variable	Grade[a]
Probability Distribution	Multinomial
Link Function	Cumulative logit

a. The procedure applies the cumulative link function to the dependent variable values in descending order.

Case Processing Summary

	N	Percent
Included	93	49.5%
Excluded	95	50.5%
Total	188	100.0%

Categorical Variable Information

			N	Percent
Dependent Variable	Grade	3	27	29.0%
		2	26	28.0%
		1	40	43.0%
		Total	93	100.0%
Factor	Week	8	53	57.0%
		4	40	43.0%
		Total	93	100.0%
	Substrain	4C57BL/6J	20	21.5%
		3C57BL/6N	25	26.9%
		2BALB/cJ	25	26.9%
		1BALB/cN	23	24.7%
		Total	93	100.0%
	Sex	M	38	40.9%
		F	55	59.1%
		Total	93	100.0%

Goodness of Fit[a]

	Value	df	Value/df
Deviance	20.898	18	1.161
Scaled Deviance	20.898	18	
Pearson Chi-Square	19.083	18	1.060
Scaled Pearson Chi-Square	19.083	18	
Log Likelihood[b]	-28.293		
Akaike's Information Criterion (AIC)	84.586		

Finite Sample Corrected AIC (AICC)	89.971		
Bayesian Information Criterion (BIC)	120.042		
Consistent AIC (CAIC)	134.042		

Dependent Variable: Grade
Model: (Threshold), Week, Substrain, Sex, Week * Substrain, Substrain * Sex, Week * Sex[a]
a. Information criteria are in smaller-is-better form.
b. The full log likelihood function is displayed and used in computing information criteria.

Omnibus Test[a]

Likelihood Ratio Chi-Square	df	Sig.
62.017	12	.000

Dependent Variable: Grade
Model: (Threshold), Week, Substrain, Sex, Week * Substrain, Substrain * Sex, Week * Sex[a]
a. Compares the fitted model against the thresholds-only model.

Tests of Model Effects

	Type III		
Source	Wald Chi-Square	df	Sig.
Week	32.380	1	.000
Substrain	2.206	3	.531
Sex	.038	1	.845
Week * Substrain	10.650	3	.014
Substrain * Sex	3.816	3	.282
Week * Sex	1.193	1	.275

Dependent Variable: Grade

Model: (Threshold), Week, Substrain, Sex, Week * Substrain, Substrain * Sex, Week * Sex

Parameter Estimates

Parameter		B	Std. Error	95% Wald Confidence Interval Lower	95% Wald Confidence Interval Upper	Hypothesis Test Wald Chi-Square	Hypothesis Test df	Hypothesis Test Sig.
Threshold	[Grade=3]	-3.142	.9591	-5.022	-1.263	10.734	1	.001
	[Grade=2]	-1.037	.8939	-2.789	.715	1.345	1	.246
[Week=8]		-2.766	1.0249	-4.775	-.757	7.284	1	.007
[Week=4]		0[a]	.	.	.	.	.	.
[Substrain=4C57BL/6J]		2.233	1.8004	-1.296	5.762	1.538	1	.215
[Substrain=3C57BL/6N]		1.342	1.4307	-1.462	4.146	.880	1	.348
[Substrain=2BALB/cJ]		-.564	1.1448	-2.808	1.680	.243	1	.622
[Substrain=1BALB/cN]		0[a]	.	.	.	.	.	.
[Sex=M]		.940	1.2008	-1.414	3.293	.612	1	.434
[Sex=F]		0[a]	.	.	.	.	.	.
[Week=8] * [Substrain=4C57BL/6J]		-3.820	1.7766	-7.302	-.338	4.623	1	.032
[Week=8] * [Substrain=3C57BL/6N]		-2.154	1.4753	-5.045	.738	2.131	1	.144
[Week=8] * [Substrain=2BALB/cJ]		1.028	1.2622	-1.446	3.502	.663	1	.415
[Week=8] * [Substrain=1BALB/cN]		0[a]	.	.	.	.	.	.
[Week=4] * [Substrain=4C57BL/6J]		0[a]	.	.	.	.	.	.
[Week=4] * [Substrain=3C57BL/6N]		0[a]	.	.	.	.	.	.
[Week=4] * [Substrain=2BALB/cJ]		0[a]	.	.	.	.	.	.
[Week=4] * [Substrain=1BALB/cN]		0[a]	.	.	.	.	.	.
[Substrain=4C57BL/6J] * [Sex=M]		-2.543	1.6952	-5.865	.780	2.250	1	.134

[Substrain=4C57BL/6J] * [Sex=F]	0[a]	.	.	.	.	.	.
[Substrain=3C57BL/6N] * [Sex=M]	-2.485	1.4072	-5.243	.273	3.119	1	.077
[Substrain=3C57BL/6N] * [Sex=F]	0[a]	.	.	.	.	.	.
[Substrain=2BALB/cJ] * [Sex=M]	-1.458	1.2877	-3.982	1.066	1.283	1	.257
[Substrain=2BALB/cJ] * [Sex=F]	0[a]	.	.	.	.	.	.
[Substrain=1BALB/cN] * [Sex=M]	0[a]	.	.	.	.	.	.
[Substrain=1BALB/cN] * [Sex=F]	0[a]	.	.	.	.	.	.
[Week=8] * [Sex=M]	1.147	1.0504	-.911	3.206	1.193	1	.275
[Week=8] * [Sex=F]	0[a]	.	.	.	.	.	.
[Week=4] * [Sex=M]	0[a]	.	.	.	.	.	.
[Week=4] * [Sex=F]	0[a]	.	.	.	.	.	.
(Scale)	1[b]						

Dependent Variable: Grade

Model: (Threshold), Week, Substrain, Sex, Week * Substrain, Substrain * Sex, Week * Sex

a. Set to zero because this parameter is redundant.

b. Fixed at the displayed value.
